# Investigating the Thermal-Protective Performance of Fire-Retardant Fabrics Considering Garment Aperture Structures Exposed to Flames

**DOI:** 10.3390/ma13163579

**Published:** 2020-08-13

**Authors:** Miao Tian, Qi Wang, Yiting Xiao, Yun Su, Xianghui Zhang, Jun Li

**Affiliations:** 1 Key Laboratory of Clothing Design and Technology, Donghua University, Ministry of Education, Shanghai 200051, China; tianmiao@dhu.edu.cn (M.T.); zhangxianghui@dhu.edu.cn (X.Z.); 2 College of Fashion and Design, Donghua University, Shanghai 200051, China; 2191173@mail.dhu.edu.cn (Q.W.); 160610225@mail.dhu.edu.cn (Y.X.)

**Keywords:** fire-retardant fabrics, thermal protective performance, air gap, garment apertures, thermal aging

## Abstract

The application of fire-retardant fabrics is essential for providing thermal protective function of the garments. Appropriate clothing design are beneficial for preventing the wearers from skin burn injuries and heat strains simultaneously. The intention of this work was to investigate the effects of clothing ventilation designs on its thermal protective performance by bench-scale tests. Four boundary conditions were designed to simulate the garment aperture structures on fabric level. Tests of thermal shrinkage, mass loss and time-to-second-degree-burns were performed with and without air gap under three heat-flux levels for two kinds of inherently fire-retardant fabrics. The impacts of fabric type, heat-flux level, air gap and boundary condition were analyzed. The presence of a 6.4-mm air gap could improve thermal protective performance of the fabrics, however, the garment openings would decrease this positive effects. More severe thermal aging found for spaced test configuration indicated the importance of balancing the service life and thermal protective performance of the clothing. The findings of this study implied that the characteristics of fabric type, air gap, boundary condition, and their effects on fabric thermal aging should be considered during clothing ventilation designs, to balance the thermal protection and comfort of the protective gear.

## 1. Introduction

Statistics from the National Fire Protection Association (NFPA) indicated that there were 58,250 estimated firefighter injuries occurred in the U.S. in 2018 [1]. Thirty nine percent of the firefighters were injured at fireground operations, which was much higher than other types of duties [2]. As the necessary personal protective equipment (PPE) [3], firefighters’ clothing provides protection for firefighters from injuries and fatalities caused by flame, radiant heat, hot surface contact, molten substances, hot liquids and steam during operations [4,5]. A variety of studies have indicated that the thermal protective performance of firefighters’ gear is complicatedly influenced by the factors of environment and garment.

The type and intensity of heat source determined the heat-transfer modes on fabric surface. Higher density convective exposure was more likely to affect the surface properties of the fabrics, leading to more severe thermal oxidation and decomposition than purely radiant exposure [6,7,8]. The combined effects of the moisture in the fabric and the heat source further influenced the heat and mass transfer within the clothing system [9,10]. Moisture in clothing system slowed fabric heating under intense radiant or convective exposures [7] and increased the thermal protective performance significantly under low radiant exposures [11,12].

As noted by Crown et al. [13], thermal protection is a function of garment design and total garment assembly as well as fiber properties and fabric structure. Flame resistant fabrics provided radiant heat protection and flame resistance are essential for firefighters’ clothing. Two categories of fire-retardant fibers commonly used in flame resistant fabrics are chemically modified fire-retardant fibers and inherently fire-retardant fibers [14]. Flame retardant materials can be applied on a substrate fiber (natural or synthetic fibers) as an additive or a finishing agent to product the chemically modified fire-retardant fibers with high pyrolyzing temperature or glass transition temperature [15,16]. However, the fire-retardant property of this kind of fibers is not stable for a long duration and toxic gases may evolve under radiant heat and/or flame [17,18]. On the other hand, the characteristics of molecular structure provide high thermal stability to the inherently fire-resistant fibers [19,20]. Therefore, the fabrics made by inherently fire-resistant fibers are commonly used as the outer layer and thermal liner of the thermal-protective clothing, such as aramid, polyimide and polybenzimidazole (PBI) [19,21,22], which are also applied in this study. The thickness and weight of the outer-shell fabrics had great positive effects on the thermal protective performance, however, the bulky protective clothing would also lead to heat stress and minimize the firefighter operational performance [6,12].

Previous studies also focused on the distribution and heat-transfer mechanism of air gap [8,23,24,25]. With the increase of exposure duration and intensity, degradative losses in thickness and mass of the fabrics occurred and the thermal shrinkage was observed for aramid fabrics [7,10,26]. Thermal shrinkage reduced the air gap width between the clothing and human skin and affected the thermo-physical properties of the fabrics [27]. The width of air gap and position have been demonstrated to play an important role on thermal protective performance [8,28,29]. Larger air gap width within certain limits and the air space position farther from heat source could produce higher thermal protective performance of the fabrics [8,30,31]. Shape memory alloy (SMA) springs in a multilayer protective fabric assembly which adjusted the structure of garment by creating an intelligent air gap between the adjacent fabric layers was also studied in recent years [32,33].

Bench-scale and full-scale manikin tests are two methods to evaluate the thermal protective performance of fire-resistant fabrics and garments, respectively. Bench-scale test developed by Behnke [34] simulated the thermal radiation and convection at fireground by using quartz tubes and Meker burners, to analyze the performance of fabric specimen based on Stoll criteria [35]. Full-scale manikin test can present the actual exposure condition encountered by the firefighters [10,35,36]. The comparative study of these two methods indicated that the bench-scale tests could represent the body regions with zero and small air gaps of the manikin tests [37]. However, the air gap changes due to thermal shrinkage of fabrics were ignored in bench-scale tests, resulting in a weak correlation with the body regions with large air gaps of the manikin tests [35]. In addition, the garment design features such as pocket flaps, collars and fabric edges were more susceptible to ignition than flat areas of the clothing system. Fabric folds, seams and wrinkles affected fabric thickness also had influences on local thermal protective performance of the garment [35,37].

For firefighters and industrial workers who operate in thermal environments, heat strain may occur as a result of the personal protective clothing being worn [38]. In addition to providing protection from external heat sources, thermal-protective clothing should be designed to minimize metabolic heat stress and provide acceptable levels of mobility and range of motion of the human body [39]. The presence of ventilation features (vents or apertures) within a clothing system has the potential to decrease excess body heat and improve the comfort [40]. However, thermal protection is of utmost importance for the firefighters [41]. Therefore, it is critical to maintain protective performance of the clothing when conducting clothing ventilation design. McQuerry et al. [42] reviewed the research on the ventilation of firefighter turnout gear including active-ventilation strategies, the effect of different moisture-barrier materials and the opening of garment cuffs to reduce heat strain. They indicated that it is essential to balance the thermal protection and the amount of heat buildup within the turnout suit and determine which types of ventilation designs are most effective for protective clothing.

The main aim of this work is to explore the influence of ventilation designs on thermal protection of the fire-retardant fabrics. A series of bench-scale tests were performed considering the garment apertures structure. The effects of four boundary conditions to simulate the garment openings were investigated for two types of fabrics, with the consideration of two air gap conditions and three heat-flux levels. Thermal shrinkage, mass loss, thermal protective performance and their correlations under the test conditions were analyzed. The findings of this study may provide references for the structure design, fabric selection and service environment determination of the thermal-protective clothing.

## 2. Materials and Methods

### 2.1. Flame-Retardant Fabrics

Two kinds of inherently fire-retardant fabrics, Nomex^®^ IIIA (DuPont, Wilmington, DE, USA) and Kevlar^®^/PBI (IBENA, Bocholt, Germany), commonly used as the outer layer of firefighter protective clothing were selected in this study. The detail characteristics of these two fabrics are shown in Table 1.

### 2.2. Test Conditions and Protocols

**Figure 1:**
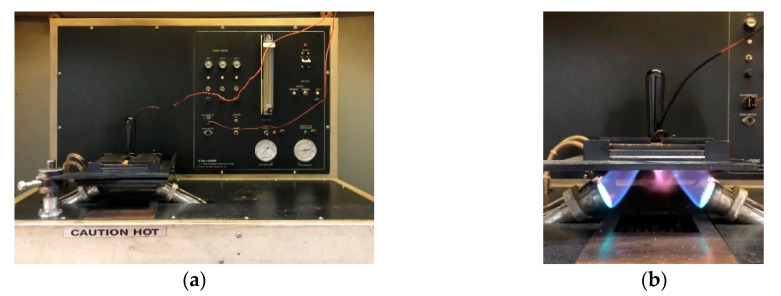
Test apparatus employed in this study: (**a**) thermal protective performance (TPP) tester; (**b**) TPP tester during flame experiment.

The test apparatus (Figure 1) employed in this study was CSI-206 (Custom Scientific Instrument Inc., Easton, PA, USA). The tester consisted of two Meker burners and nine heated quartz tubes which can produce a nominal heat flux of 84 ± 2 kW/m^2^, including 50% radiant and 50% convective heat flux. A copper calorimeter was used to measure the temperature at back of the specimen. The copper face was blackened to obtain the similar emissivity with human skin and mounted on an insulating board. The insulating board was either in contact with the specimen to simulate a snug fit condition, or with an air gap of 6.4 mm to simulate a standard fit condition. The temperatures recorded by the calorimeter were connected to a computer processing system to calculate the time-to-second-degree-burns and thermal protective performance (TPP) values.

#### 2.2.1. Experimental Variable

Only Meker burners were activated in this work to produce flame exposure. The fabric specimens were cut with a size of 15 cm × 15 cm in standard cases and the exposed fabric area was 100 cm^2^ in the center. Flame-exposure duration was determined after pre-experimental testing to be 17 s. This allowed measuring the time needed for second degree burns to occur while maintaining structural integrity of the specimens.

**Figure 2:**
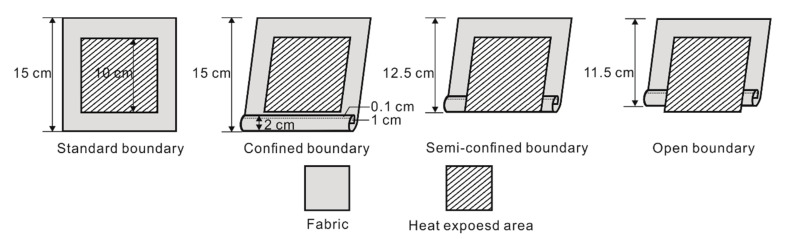
Diagram of four boundary conditions (fabric back side).

In order to simulate the garment apertures structure, four boundary conditions were designed. The coverage of skin by thermal-protective clothing at garment apertures changed with the movement of the wearers, as well as the thermal shrinkage of the fabrics, which can be divided into completely covered, sufficiently covered and partially exposed. Accordingly, three boundary conditions were designed as confined, semiconfined and open boundary. The standard boundary condition was set as the control group, as shown in Figure 2.

Two kinds of air gaps between the specimen and insulating board were employed to simulate the spaces between the clothing and human skin. A direct contact (0-mm air gap, snug fit) and a spaced configuration (6.4-mm air gap, standard fit) were designed to produce two kinds of spaces under garment. The confined and open boundary can represent the garment aperture structures with or without closures at sleeves, neck and waist, respectively. Moreover, three levels of heat flux (30 kW/m^2^, 40 kW/m^2^ and 50 kW/m^2^) were selected to explore the influence of heat-flux intensity on thermal shrinkage and thermal protective performance.

In total, there were 48 groups of experiments for two kinds of fabrics. Each group was repeated 3 times; results are given as mean values. All test specimens were preconditioned in a constant atmosphere (23 ± 2 °C and 60% ± 5% RH) for at least 24 h before testing.

#### 2.2.2. Experimental Specimens

**Figure 3:**
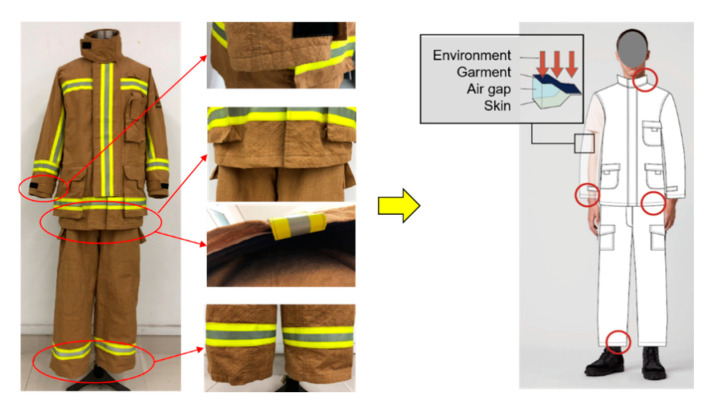
Garment aperture structures and the relationship of garment, air gap and skin.

The fabric specimens were cut and sewn based on the boundary conditions to simulate garment aperture structure as shown in Figure 3. The specimens of standard boundary were cut with the size of 15 cm × 15 cm and not folded. The test lengths of confined, semiconfined and open boundary specimens were 15 cm, 12.5 cm and 11.5 cm, respectively, as shown in Figure 2. The specimens were sewn with a 2-cm-wide hem at the bottom, in which the raw-edge-width was 1 cm. The hem was sewn with flame-retardant sewing thread matching the color of fabrics; the stitch density was 10 stitches per 3 cm.

### 2.3. Measurement and Processing of the Indicators

#### 2.3.1. Thermal Shrinkage

In order to investigate the effects of garment aperture structures on fabric thermal shrinkage, the surface area retention was calculated. The digital image technology based on the function library of OpenCV (version 3.30) was adopted to measure the area retention of the specimens. Two dimensional images were taken with a high performance scanner (Fujitsu SV600, Tokyo, Japan) prior to and after flame exposure. The images were taken directly above the specimen at the same height and then converted to black and white with the image processing program. The surface area retention was defined as:

(1) $$R=\frac{A^{\prime }}{A}\times 100\%$$

where, R is the surface area retention (%); A is the surface area of the specimen before flame exposure, m^2^; and $A^{\prime }$ is the surface area of the specimen after flame exposure, m^2^.

#### 2.3.2. Mass Loss

Fabric mass is an important impact factor on thermal protective performance of the fire-retardant fabrics, which will also change due to moisture evaporation and thermal degradation. The mass loss of the specimen was determined by weighing the fabrics immediately prior to and after flame exposure using an analytical balance with a precision of 0.1 mg. Considering the exposure-area difference for the specimen with four boundary conditions, standardized mass loss was defined as:

(2) $$M_{std}=\frac{M-M^{\prime }}{A_{exp}}\times 100\%$$

where, $M_{std}$ is the standardized mass loss, g/m^2^; *M* is the mass of the specimen before flame exposure, g; $M^{\prime }$ is the mass of the specimen after flame exposure, g; and $A_{exp}$ is the flame-exposed area of the specimen, m^2^.

### 2.4. Statistical Analysis

Mean values and standard deviations were calculated for surface area retention, standardized mass loss and time-to-second-degree-burns. An analysis of variance (ANOVA) test was carried out using SPSS version 25.0 (SPSS, Inc.) to determine whether there were any significant differences among test conditions at a significant level of 0.05. A least-significant difference (LSD) test was performed for post hoc analysis within the group. To further analyze the relationships between surface area retention, standardized mass loss and time-to-second-degree-burns, correlation analysis was employed, and the fitting curves were created for different configurations.

## 3. Results

### 3.1. Surface Area Retention

**Figure 4:**
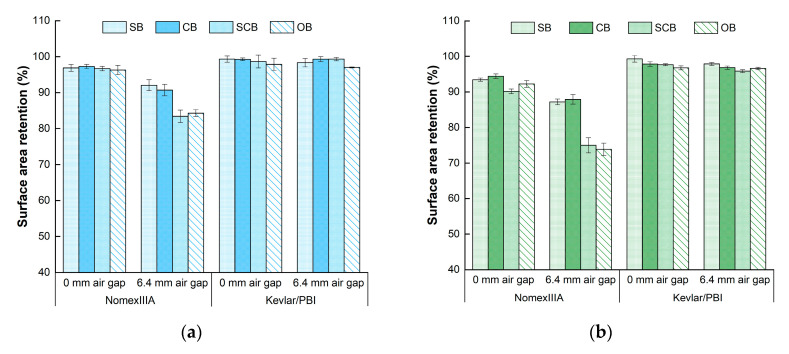

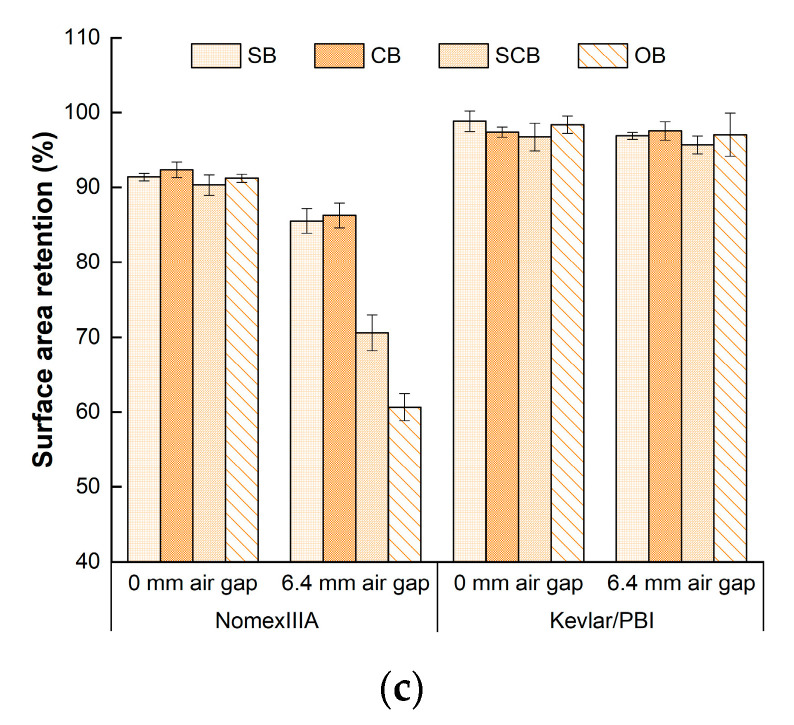
Surface area retention under the heat flux of: (**a**) 30 kW/m^2^; (**b**) 40 kW/m^2^; (**c**) 50 kW/m^2^. SB—standard boundary; CB—confined boundary; SCB—semiconfined boundary; OB—open boundary.

Figure 4 illustrates the surface area retention under different testing conditions for Nomex IIIA and Kevlar/PBI specimens. A higher surface area retention indicated better thermal stability of the fabrics. Discrepancy was observed for these two kinds of fabrics that the thermal shrinkage of Nomex IIIA was more severe than Kevlar/PBI. The surface area retention of Nomex IIIA ranged from 60.6% to 97.3%, while the values of Kevlar/PBI were between 95.7% and 99.4%. No apparent variation pattern was found for Kevlar/PBI under four boundary conditions and three heat-flux levels. The average surface area retention of Kevlar/PBI with air gap was 0.8% lower than that of direct contact configuration.

Regarding Nomex IIIA specimens, significant difference was found for surface area retention between 0-mm- and 6.4-mm-air-gap conditions (*p* < 0.001), where the average discrepancy was 12.1%. No significant difference was found for heat-flux level (*p* = 0.162) and boundary conditions (*p* = 0.254). However, the average surface area retention was decreased from 92.2% to 83.5% as the heat flux increased. The difference of the average values for standard (91.1%) and confined (91.4%) boundary conditions was minor. Relative smaller average surface area retention was observed for semiconfined boundary (84.4%), followed by open boundary condition (83.1%).

Considering the remarkable effects of air gap condition on Nomex IIIA, the significance analysis was also performed for 0-mm- and 6.4-mm-air-gap conditions separately. When there was no air gap between specimen and sensor, significant difference was detected for heat-flux level (*p* < 0.001), while the impact of boundary condition was still not significant (*p* = 0.805). A dramatically finding was that the heat flux had no significant influence on surface area retention (*p* = 0.214) under 6.4-mm-air-gap condition, which was different from the results of the 0-mm-air-gap condition. However, a declined tendency was observed as the increase of the heat-flux level, indicating that the existence of air gap would decrease the effects of heat-flux level on surface area retention. No significant difference was found for boundary conditions (*p* = 0.057), while post hoc analysis indicated significant differences between standard, confined boundary and open boundary condition (*p* = 0.029).

### 3.2. Standardized Mass Loss

**Figure 5:**
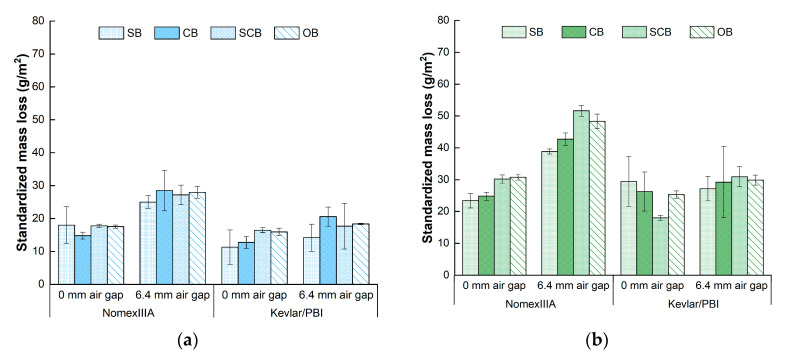

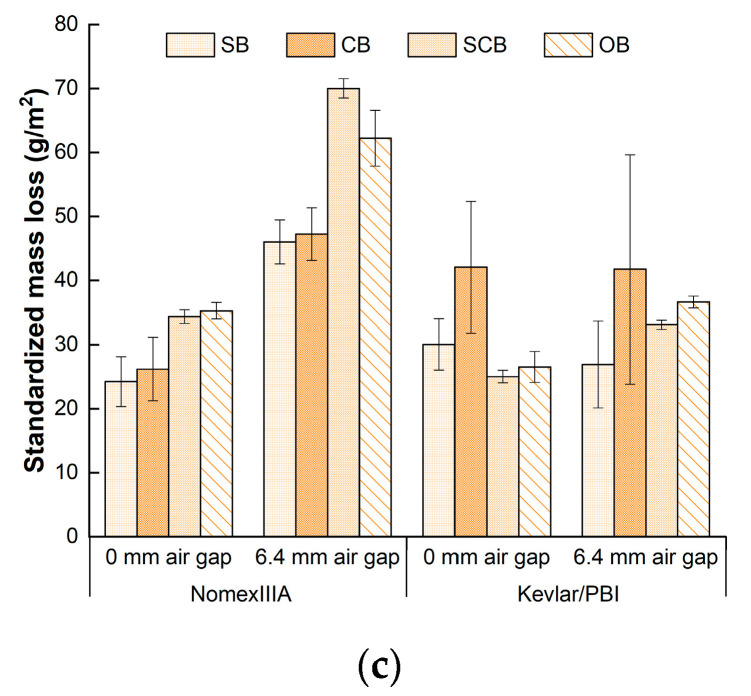
Standardized mass loss under the heat flux of: (**a**) 30 kW/m^2^; (**b**) 40 kW/m^2^; (**c**) 50 kW/m^2^. SB—standard boundary; CB—confined boundary; SCB—semiconfined boundary; OB—open boundary.

The averages and standard deviations of standardized mass loss were calculated and presented in Figure 5. It was obvious that the standardized mass loss of Nomex IIIA was greater than that of Kevlar/PBI, especially under the condition of 6.4-mm air gap. The values ranged from 14.8 g/m^2^ to 70 g/m^2^ for Nomex IIIA and from 11.3 g/m^2^ to 42.1 g/m^2^ for Kevlar/PBI. An average standardized mass loss of 24.8 g/m^2^ was found for Nomex IIIA under the condition of 0-mm air gap, which was 1.5 g/m^2^ larger than that of Kevlar/PBI. The values of these two kinds of fabrics were higher under 6.4-mm-air-gap condition, which were 43 g/m^2^ and 27.2 g/m^2^, respectively.

Significant differences were found for fabric type (*p* = 0.015), air gap (*p* = 0.001) and heat-flux level (*p* < 0.001). The average values increased from 19.0 g/m^2^ to 31.7 g/m^2^ and 38.0 g/m^2^ with the elevated heat fluxes. The impact of boundary on standardized mass loss was not significant (*p* = 0.753). Considering the fabric type separately, similar variation were found for air gap and heat flux that the existence of air gap and increased heat flux significantly enhanced the standardized mass loss. The standardized mass loss of Nomex IIIA was higher for semiconfined and open boundary compared with standard and confined boundary conditions under 40 kW/m^2^ and 50 kW/m^2^ heat flux conditions. However, the effects of boundary conditions on standardized mass loss were not obvious for Kevlar/PBI.

### 3.3. Time-to-Second-Degree-Burns

**Figure 6:**
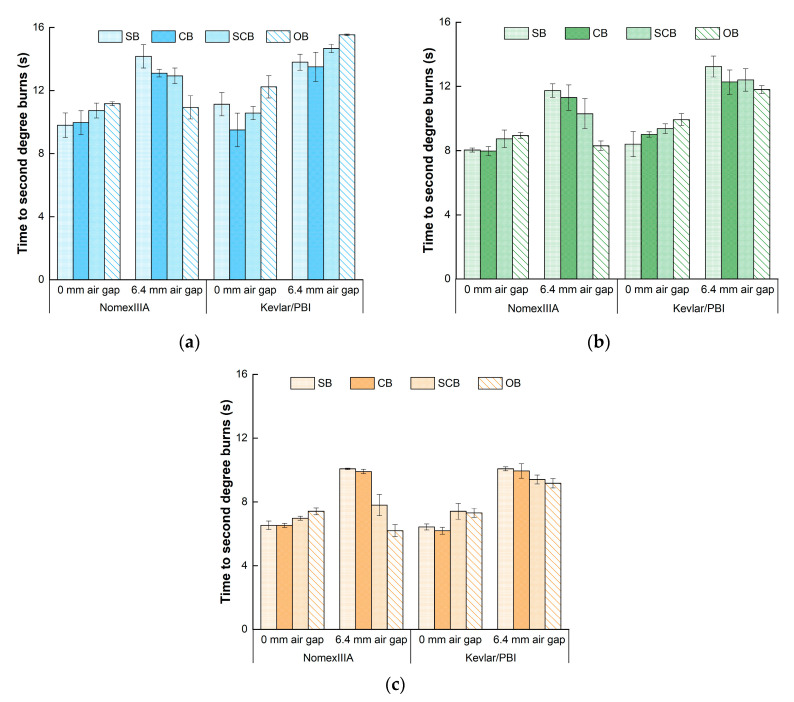
Time-to-second-degree-burns under the heat flux of: (**a**) 30 kW/m^2^; (**b**) 40 kW/m^2^; (**c**) 50 kW/m^2^. SB—standard boundary; CB—confined boundary; SCB—semiconfined boundary; OB—open boundary.

Thermal protective performance of the fire-retardant fabrics was commonly characterized by the time-to-second-degree-burns and longer time indicated better protective performance. Figure 6 displays the time-to-second-degree-burns under different testing conditions. The average time-to-second-degree-burns for Kevlar/PBI (10.6 s) was 1 second longer than Nomex IIIA (9.6 s) specimens. A larger average second degree burn time was found for the 6.4-mm-air-gap condition than the 0-mm-air-gap condition for both of Nomex IIIA and Kevlar/PBI. The values ranged from 6.5 s to 11.2 s for Nomex IIIA without an air gap and from 6.2 s to 14.2 s for the 6.4-mm air gap. The second degree burn time for Kevlar/PBI was between 6.2 s to 12.2 s for the 0-mm-air-gap condition and between 9.2 s to 15.5 s for the 6.4-mm-air-gap condition. All time-to-second-degree-burns for the spaced test configuration was higher than the contact test configuration.

One-way analysis of variance indicated significant effects of air gap (*p* < 0.001) and heat-flux level (*p* < 0.001). Time-to-second-degree-burns for 6.4-mm air gap was significant—greater than 0-mm-air-gap condition. The values decreased from 12.1 s to 10.1 s and 8.0 s with the increase of heat-flux levels. No significant differences were found for different fabric type (*p* = 0.156) and boundary conditions (*p* = 0.981).

Although there was no significant difference between Nomex IIIA and Kevlar/PBI, different tendency was found in Figure 6. Therefore, these two kinds of fabric was also analyzed separately. As for Nomex IIIA, significant effects were observed for air gap (*p* = 0.023) and heat flux (*p* < 0.001). Boundary conditions had no significant impact (*p* = 0.812). For the contact test configuration, although the influence of the boundary conditions was still insignificant (*p* = 0.862), an increased variation tendency was found for standard (8.1 s), confined (8.2 s), semiconfined (8.8 s) and open (9.2 s) boundary conditions. Note that the variation pattern for the spaced test configuration was opposite that a decrease trend was found for these four boundary conditions, with the second degree burn times of 12.0 s, 11.4 s, 10.3 s and 8.5 s. A similar trend of Nomex IIIA was detected for three levels of heat flux. The significant test for Kevlar/PBI indicated the same results with Nomex IIIA. Time-to-second-degree-burns increased with the existence of air gap and decreased as heat flux increased. However, the variation tendency for different boundary conditions was not obvious.

## 4. Discussion

Previous research indicated the necessity to balance the needs of maximize thermal protection and minimize metabolic heat stress [39]. To determine which types of ventilation designs are the most effective way to improve thermal-protective clothing [42]. Four boundary conditions were designed to simulate the garment aperture structures and the protective performance were investigated by bench-scale tests in this study. Research based on full-scale manikin tests revealed that garment shrinkage during exposure could greatly reduce the air gap and potentially cause a significant decrease in the performance of thermal-protective clothing [33,43]. Surface area changes could represent the thermal shrinkage of the fire-retardant fabrics [44]. A higher surface area retention indicated better thermal stability of the fabrics. Our study showed that the thermal stability of Kevlar/PBI was superior to Nomex IIIA, which was in accordance with the literature. Fiber type and fabric structure are two factors influencing the thermal shrinkage of the fire-retardant fabrics [25]. The internal stresses resulted from the spinning and drawing processing during the fiber formation process tend to relax, and the macromolecule chains tend to retract from extended conformation to random coil when the fabric exposed to heat source, which led to shrinkage in the length direction of the fiber [27,45]. The shrinkage was directly proportional to temperature increases [43,46]. As for Nomex and PBI fiber, the difference of glass transition temperature (T_g_) and crystallinity determined the different shrinkage behavior of the fabrics [47,48].

The result of surface area retention also demonstrated that thermal shrinkage of Nomex IIIA was more severe under 6.4-mm-air-gap condition compared with contact test configuration. The main reason was that the sensor insulating board was on top of the specimen, which produced a uniform pressure to restrict the shrinkage of the fabrics. However, there were space between the fabric and sensor insulating board for the spaced test configuration, and the forces produced by the instrument only presented on the edges of the specimen. Besides, Li et al. [43] established a correlation between the air gap size and the thermal shrinkage of the garment based on flame manikin tests and found a Spearman correlation coefficient of 0.749 with confidence level of 0.01. This research also indicated that thermal shrinkage was fundamentally related to the force sustained by the fabric and garment due to the body geometry, garment design features and fabric mechanical properties. The purpose of this study to design semiconfined and open boundary conditions was to simulate clothing openings and compared with the standard condition. Apparently, the thermal shrinkage under these two boundary conditions was greater than the standard and confined boundary conditions, which were also related to the forces applied to the edges of the specimens. Regarding the standard and confined boundary conditions, four edges of the fabric were fixed, however, only three edges were fixed for semiconfined and open boundary conditions. This result was in line with the finding of Li et al. [43] that cuff and leg hems of the garments were easily shrunk during exposure to flash fire due to loose structure without restriction.

**Figure 7:**
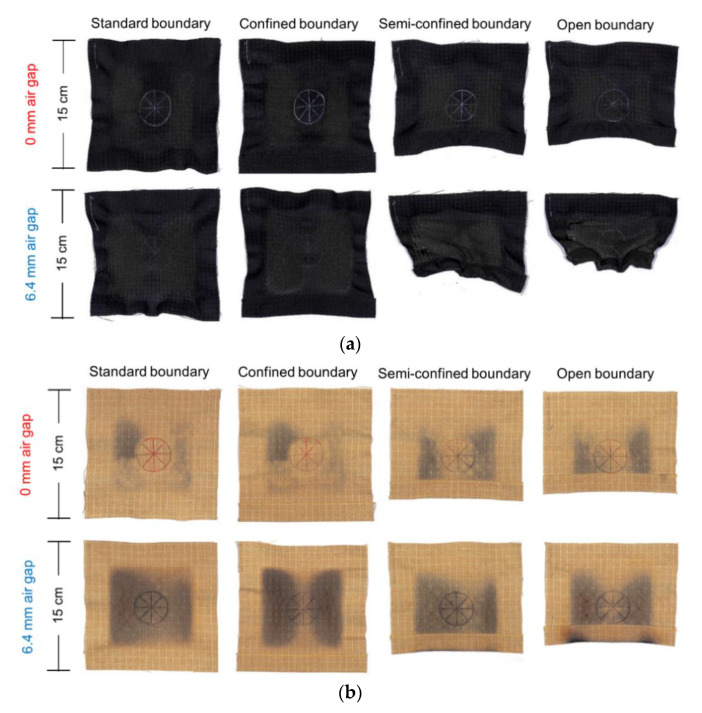
Backside photographs of the specimens after fire exposure under the heat flux of 50 kW/m^2^: (**a**) Nomex IIIA specimens. Thermal shrinkage was more severe in the presence of air gap for semiconfined and open boundary conditions; (**b**) Kevlar/PBI specimens. No significant thermal shrinkage of the specimens, while the area of color fading and carbonization is valid evidenced of the effect of air gap.

Photographs shown in Figure 7 demonstrate the more severe thermal shrinkage of Nomex IIIA and the negative effects of the air gap. The conditions of semiconfined and open boundary also enhanced the shrinkage of Nomex IIIA, especially at the free boundary edge. Wrinkles and folds were generated for Nomex IIIA. According to Figure 7, both of Nomex IIIA and Kevlar/PBI specimens experienced significant color fading and carbonization. Under the condition of 6.4-mm air gap, the area of color fading and carbonization was larger for all boundary conditions compared with no air gap condition, which was verified by the large black area of the specimens. The appearance change of the specimens can also explained the variation of standardized mass loss.

As noted by Barker et al. [7], the mass loss induced by heat of the flame-retardant fabrics was mainly through moisture evaporation and by evolution of volatile degradation products. Direct flame contact of the fabric surface used in this study produced a more severe condition for thermal oxidation and decomposition of surface fibers than produced by purely radiant exposures. Larger standardized mass loss was found for Nomex IIIA than Kevlar/PBI specimens in this study. The existence of 6.4-mm air gap also exacerbated the mass loss of the fabrics, which can be explained by the more severe discoloration and carbonization shown in Figure 7. Moreover, the standardized mass loss of the specimens increased with the elevated heat-flux levels, which was in accordance with the findings of Barker et al. [7] that the severity of the surface degradation and loss of surface fibers can be explained by a higher heating rate.

Second degree burn time is a typical index to represent the thermal protective performance of the flame-retardant fabrics. Although with lower mass and thickness, larger average time-to-second-degree-burns was observed for chosen Kevlar/PBI fabrics, where the more severe thermal shrinkage of the Nomex IIIA specimens was probably the main cause. The presence of air gap also improved thermal protective performance of the specimens, which was related to the insulation of air layer and the heat-transfer modes. Torvi [49] investigated the effect of air space between fabric and sensor on thermal protection and found that the air gap size played an important role in the results of benchtop tests of thermal protective fabrics. Sawcyn [50] also indicated that second degree skin burn time increased with the increasing of air gaps obtained experimentally using single open flame heat source of 80 kW/m^2^.

**Figure 8:**
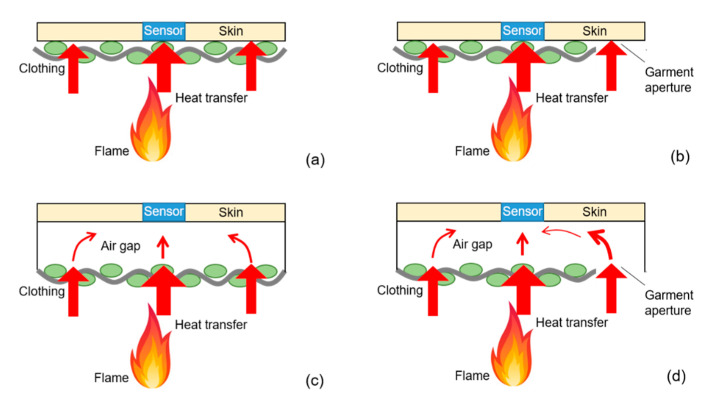
Schematic diagram of the heat-transfer process. (**a**) Standard/confined boundary condition without air gap; (**b**) open boundary condition without air gap; (**c**) standard/confined boundary condition with air gap; (**d**) open boundary condition with air gap.

Figure 8 illustrates the heat-transfer process for direct contact and spaced test configurations under the conditions of standard/confined and open boundary. Regarding the contact test configuration shown in Figure 8a, the sensor insulating board was placed in direct contact with the specimen. Heat generated by the flame transferred to the specimen by thermal radiation, convection and conduction, increasing the temperature of the fabric. The energy stored in the fabric was mainly transferred to the sensor by conduction. Therefore, the basic and thermophysical properties (such as thickness and thermal conductivity) of the fabric were the controlling factor determining heat-transfer rate. As for the spaced test configuration displayed in Figure 8c, the presence of the air layer with good thermal insulation decreased the heat-transfer rate to the sensor. A large amount of energy was stored in the fabric before it was transferred to the sensor [51], resulting in the temperature rise and degeneration of the specimen. According to the research of Wang et al. [8], energy transferred to inner layer decreased due to low thermal conductivity of air for multilayered fabric system. The damage of inner layer was much severe when the air gap located between inner layer and sensor insulating board, than that produced at any other position near to the out shell fabric. This can also explain the more severe thermal shrinkage, discoloration and carbonization, as well as greater mass loss of the specimens under the spaced test configuration compared with the direct contact configuration in this study. On the other hand, the existence of 6.4-mm air gap could decrease heat-transfer rate to the sensor, increase the time-to-second-degree-burns, hence improve the thermal protective performance of the fabrics, as shown in Figure 6.

Figure 8b illustrated the heat-transfer process for contact test configuration with garment aperture. Variance analysis indicated that the boundary condition had no significant effects on surface area retention, standardized mass loss and time-to-second-degree-burns under 0-mm-air-gap condition. Although there was garment aperture as shown in Figure 8b, the heat source and heat-transfer mode was similar with Figure 8a. Energy of the flame could transfer to the sensor insulating board directly through the garment aperture, however, the copper sensor was embedded in the center of the board, which was barely affected. Nonetheless, an escalating trend was found for time-to-second-degree-burns from standard to confined, semiconfined and open boundary condition under the contact test configuration. The possible reason was that the sewn hem increased the thickness of the bottom edge of the specimen and produced a tiny air layer between the fabric and sensor, which increased the thermal protective of the specimen. The heat-transfer mechanism for the spaced test configuration was much more complicated, especially for the open boundary condition with garment aperture shown in Figure 8d. Heat partially transferred from burners to the sensor by heat conduction of air, radiation transfer induced by heated fabric, and the radiation energy penetrating through the fabric. Garment aperture was another channel for heat transfer from the burner to the air layer and to the sensor. Therefore, the open boundary condition decreased the thermal protective performance of the fabrics.

**Figure 9:**
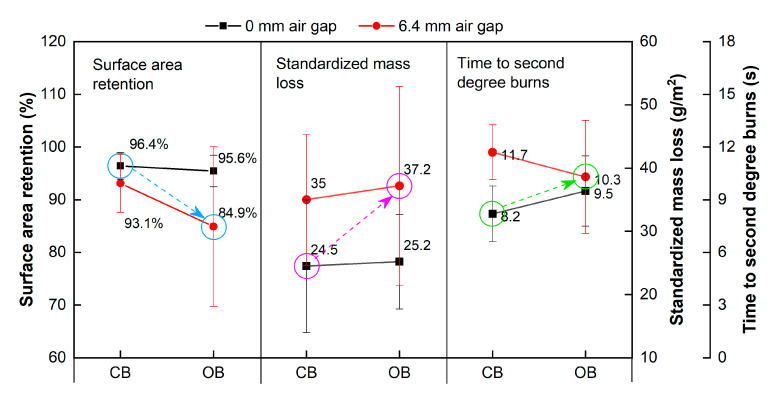
Comparison of the results between confined and open boundary conditions. CB—confined boundary; OB—open boundary.

Considering the confined and open boundary can represent the garment aperture structures with or without closures at sleeves, neck and waist, respectively, their effects on surface area retention, standardized mass loss and time-to-second-degree-burns were compared in Figure 9. Apparently, the presence of 6.4-mm air gap enhanced the thermal shrinkage and mass loss, which accelerated the thermal aging and declined the lifetime of the flame-retardant fabrics for both of confined and open boundary conditions. On the contrary, thermal protective performance of the fabrics were improved due to the existence of the 6.4-mm air gap, indicating the importance of balancing the service life and thermal protective performance of the thermal-protective clothing. Compared the snug fit to the standard fit condition, garment without closures at sleeves, neck and waist would significantly increase the thermal shrinkage and mass loss. However, the garment openings would decrease the positive effect of air gap on thermal protective performance. According to Rucker et al. [39], the loose-fitting garments provided a significantly higher level of burn protection. However, they also cautionary noted that garment looseness must be controlled with closures that allowed a snug fit at the collar, cuffs and waist, to avoid the chimney effects on greater injuries.

**Figure 10:**
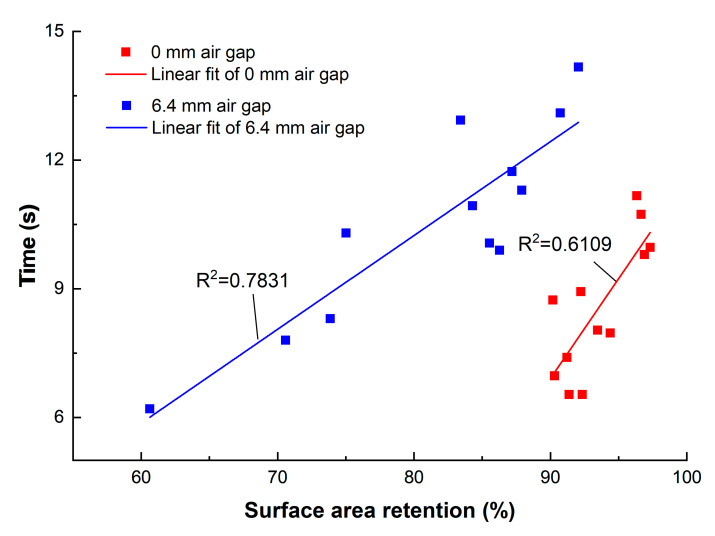
Relationship between the surface area retention and thermal protective performance under 0-mm- and 6.4-mm-air-gap conditions of Nomex IIIA specimens.

To further understand the correlations of time-to-second-degree-burns with surface area retention and standardized mass loss, correlation analysis was performed. Considering the good thermal stability of the Kevlar/PBI fabrics, the results of surface area retention for Nomex IIIA specimens were applied to perform the correlation analysis, which is shown in Figure 10. The correlation coefficients were 0.7816 and 0.885 for the 0-mm- and the 6.4-mm-air-gap condition, respectively, and the significance level were both less than 0.001. Equations (3) and (4) indicated the relationship between time-to-second-degree-burns and surface area retention for two test configurations, with the R^2^ of 0.6109 and 0.7831, respectively. The positive linear correlations indicated that time-to-second-degree-burns grew with the increase of surface area retention. This meant that the thermal protective performance of the flame-retardant fabrics would be improved when thermal shrinkage of the fabrics was small.

(3) $$t_{contact}=0.4657x-35$$

(4) $$t_{spaced}=0.2188x-7.26$$

The relationship of standardized mass loss and time-to-second-degree-burns was also analyzed and the results were presented in Table 2. The fitting curves of the four boundary conditions were established separately. As for the contact test configuration, significant negative correlations were found with the Pearson’s correlation coefficient of −0.8432, −0.8418, −0.8414 and −0.8739 for standard, confined, semiconfined and open boundary condition (*p* < 0.05). Moreover, all of the R^2^ of the fitting curves were over 0.7. Negative correlations were also observed for the spaced test configuration. The correlation analysis showed that time-to-second-degree-burns declined with the standardized mass loss increased. The mass loss is mainly through moisture evaporation and degradation, and the moisture evaporation is inevitable when the fabrics exposed to heat source. Therefore, decelerating the thermal aging progress will be a potential approach to maintain the thermal protective performance of the flame-retardant fabrics.

According to Rucker et al. [39] and McQuerry et al. [42], the function of thermal-protective clothing was to provide protection against external thermal threats to prevent burn injuries and transfer internally generated heat to minimize heat stress injuries experienced by the wearer. Proper garment ventilation design is one potential avenue to achieve this purpose and should be explored [42]. In this study, we mainly focused on the thermal protective performance of the fire-retardant fabrics considering the garment aperture design and discussed its different effects of direct contact and spaced test configurations. Results also demonstrated the thermal aging of the fire-retardant fabrics based on the thermal shrinkage, mass loss and appearance change, influencing the useful life of the fabrics. Previous studies reported that the high-performance materials used to manufacture fire protective clothing are sensitive to the environmental agents to which the clothing is exposed in service [52]. The decomposition reaction temperature ranged from 425 °C to 625 °C for Nomex IIIA and from 550 °C to 650 °C for Kevlar/PBI [53]. Elevated temperature exposures tended to induce large decreases in the mechanical performance of yarns and fabrics [52]. The thermal aging of Nomex fibers was shown to result in a reduction in the fiber elastic modulus, tensile strength and elongation at break [54]. The thermal aging of Kevlar/PBI blends was attributed to the change of a crystallite size crystalline lattice of Kevlar and random chain scissions of PBI [55]. A full-scale flame manikin study revealed the complicated effects of garment structure on thermal degradation of thermal-protective clothing [56]. Numeric simulation also indicated that the heat transfer in the air space was more complicated on the garment level than the fabric, because of the varying relative positions between different body segments and the heat source, as well as the ventilation openings [57]. It is vital to balance the protective function and comfort of the thermal-protective clothing, simultaneously with prolonging its service life. Designing fire protective gear after fully considered the thermal conditions that will be experienced by the clothing [58] and combining the configuration of fire-retardant fabrics and design of garment pattern, is necessary for improving the usability of the protective gear.

## 5. Conclusions

Thermal protective gear is an important barrier to protect firefighters or industrial workers from thermal hazards by providing insulation against heat transmission to human skin. Appropriate clothing design and scientific using guidelines of thermal-protective clothing are beneficial for preventing the wearers from skin burn injuries and heat strains simultaneously. Considering the potential of adopting the ventilation designs to balance the protection and heat loss of the protective gear, four boundary conditions were designed in this study to simulate the garment aperture structures at sleeves, pants and lower hem. The main conclusions are drawn as follows:

(1) Significant effects of heat flux on standardized mass loss and time-to-second-degree-burns were observed. Heat flux had no significant influence on surface area retention under 6.4-mm-air-gap condition for Nomex IIIA, which was different from the results of 0-mm-air-gap condition, indicating that the existence of air gap would decrease the effects of heat-flux level on surface area retention;
(2) The impacts of boundary condition were not remarkable on fabric level, where the limitation of the bench-scale tester was the main reason. However, significant differences of surface area retention were found between standard, confined boundary and open boundary condition;
(3) Air gap had significant effects on surface area retention, standardized mass loss and time-to-second-degree-burns. Although the presence of 6.4-mm air gap could improve the thermal protective performance of the fabrics, more severe thermal shrinkage, discoloration, carbonization and mass loss were detected compared with the direct contact configuration, which accelerated the thermal aging and declined the lifetime of the flame-retardant fabrics, indicating the importance of balancing the service life and thermal protective performance of the thermal-protective clothing;
(4) Time-to-second-degree-burns grew with the increase of surface area retention, implying that the thermal protective performance was better for the flame-retardant fabrics with less thermal shrinkage. Negative correlations between time-to-second-degree-burns and standardized mass loss indicated that decelerating the thermal aging progress will be a potential approach to maintain the thermal protective performance of the flame-retardant fabrics.

The findings of this study indicated that the characteristics of fabric type, air gap, boundary condition, as well as their effects on thermal aging of the fabrics should be considered during ventilation designs of the protective gear.

## Figures and Tables

**Table 1:** Basic properties of the specimens.

Fabric	Component	Weave Type	Mass (g/m^2^)	Thickness (mm)	Color
Nomex IIIA	93% meta-aramid/5% para-aramid/2% anti-static fiber	Plain	240	0.50	Dark blue
Kevlar/PBI	60% para-aramid/40% meta-aramid	Plain	200	0.34	Dark yellow

**Table 2:** Pearson’s correlation coefficient and R^2^ of the fitting curves.

Condition	0-mm Air Gap				6.4-mm Air Gap			
	SB	CB	SCB	OB	SB	CB	SCB	OB
*r*	−0.8432	−0.8418	−0.8414	−0.8739	−0.6791	−0.9362	−0.8710	−0.9409
*p*	0.035 *	0.036 *	0.036 *	0.023 *	0.138	0.006 **	0.024 *	0.005 **
R^2^	0.7110	0.7086	0.7080	0.7637	0.4612	0.8765	0.7586	0.8853

SB—standard boundary; CB—confined boundary; SCB—semiconfined boundary; OB—open boundary; *r* is the Pearson’s correlation coefficient; *p* is the significance level; * *p* < 0.05; ** *p* < 0.01.

## References

[1] Campbell R., Molis J.L. (2019). U.S. Firefighter injuries in 2018. NFPA J..

[2] Fahy R.F., Molis J.L. (2018). Firefighter Fatalities in the US-2018. National Fire Protection Association.

[3] Scott R.A. (2005). Textiles for Protection.

[4] Abbott N.J., Schulman S. (1976). Protection from fire: Nonflammable fabrics and coatings. J. Coat. Fabr..

[5] Barker R.L. (2005). A Review of Gaps and Limitations in Test Methods for First Responder Protective Clothing and Equipment: National Personal Protection Technology Laboratory.

[6] Lee Y.M., Barker R.L. (1987). Thermal protective performance of heat-resistant fabrics in various high intensity heat exposures. Text. Res. J..

[7] Barker R.L., Lee Y.M. (1987). Analyzing the transient thermophysical properties of heat-resistant fabrics in TPP exposures. Text. Res. J..

[8] Wang Y.Y., Lu Y.H., Li J., Pan J.H. (2012). Effects of air gap entrapped in multilayer fabrics and moisture on thermal protective performance. Fibers Polym..

[9] Lawson L.K., Crown E.M., Ackerman M.Y., Dale J.D. (2004). Moisture effects in heat transfer through clothing systems for wildland firefighters. Int. J. Occup. Saf. Ergon..

[10] Talukdar P., Das A., Alagirusamy R. (2016). Heat and mass transfer through thermal protective clothing—A review. Int. J. Therm. Sci..

[11] Song G.W., Paskaluk S., Sati R., Crown E.M., Doug Dale J., Ackerman M. (2011). Thermal protective performance of protective clothing used for low radiant heat protection. Text. Res. J..

[12] Lee Y.M., Barker R.L. (1986). Effect of moisture on the thermal protective performance of heat-resistant fabrics. J. Fire Sci..

[13] Crown E.M., Ackerman M.Y., Dale J.D., Tan Y. (1998). Design and evaluation of thermal protective flightsuits. Part II: Instrumented mannequin evaluation. Cloth. Text. Res. J..

[14] Song G.W., Mandal S., Rossi R. (2016). Thermal Protective Clothing for Firefighters.

[15] Horrocks A.R., Heywood D.H. (2003). Flame retardant finishes and finishing. Textile Finishing, Society of Dyers and Colorists.

[16] Kowbel W., Patel K., Withers J.C., Repecka L., Saremi F.F. (2001). Fire resistant coating for polymeric fibers. A Materials and Processes Odyssey, SOC Advancement Material and Process Engineering.

[17] Horrocks A.R., Tune M., Cegielka L. (2010). The burning behaviour of textiles and its assessment by oxygen-index methods. Text. Prog..

[18] Jones W.W. (1985). A multicompartment model for the spread of fire, smoke and toxic gasses. Fire Saf. J..

[19] Tsvetkov V.N., Shtennikova I.N. (1978). Structure and Rigidity of Molecules of Aromatic Polyamides in Solutions. Macromolecules.

[20] Yoda N. (1962). Studies of the structure and properties of aromatic polyamide. I. Physical properties of poly (m-xylyleneadipamide). Bull. Chem. Soc. Jpn..

[21] Lawson J.R. (1997). Fire Fighters’ Protective Clothing and Thermal Environments of Structural Fire Fighting in Performance of Protective Clothing: Sixth Volume.

[22] Hearle J.W.S. (2001). High Performance Fibres.

[23] Li X., Wang Y., Lu Y. (2011). Effects of body postures on clothing air gap in protective clothing. J. Fiber Bioeng. Inform..

[24] Ghazy A. (2014). Numerical study of the air gap between fire-protective clothing and the skin. J. Ind. Text..

[25] Takada S., Sasaki A., Kimura R. (2016). Fundamental study of ventilation in air layer in clothing considering real shape of the human body based on cfd analysis. Build. Environ..

[26] Wang C.C., Chen C.C. (2005). Nylon-6 with a short rigid central block. J. Appl. Polym. Sci..

[27] Wang M., Li X.H., Li J., Xu B. (2015). A new approach to quantify the thermal shrinkage of fire protective clothing after flash fire exposure. Text. Res. J..

[28] Li X.H., Lu Y.H., Li J., Wang Y.Y., Zhou L. (2015). A new approach to evaluate the effect of moisture on heat transfer of thermal protective clothing under flashover. Fiber Polym..

[29] Fu M., Weng W., Yuan H. (2014). Effects of multiple air gaps on the thermal performance of firefighter protective clothing under low-level heat exposure. Text. Res. J..

[30] Talukdar P., Das A., Alagirusamy R. (2017). Numerical modeling of heat transfer and fluid motion in air gap between clothing and human body: Effect of air gap orientation and body movement. Int. J. Heat Mass Tran..

[31] Song G.W. (2007). Clothing air gap layers and thermal protective performance in single layer garment. J. Ind. Text..

[32] He J.Z., Lu Y.H., Wang L.J., Ma N.N. (2018). On the improvement of thermal protection for temperature-responsive protective clothing incorporated with shape memory alloy. Materials.

[33] Ma N.N., Lu Y.H., He J.Z., Dai H.Q. (2018). Application of shape memory materials in protective clothing: A review. J. Text. Ind..

[34] Behnke W.P. (1997). Thermal protective performance test for clothing. Fire Technol..

[35] Wang M., Li X.H., Li J. (2015). Correlation of bench scale and manikin testing of fire protective clothing with thermal shrinkage effect considered. Fiber Polym..

[36] Wang Y.Y., Wang Z.L., Zhang X., Wang M., Li J. (2015). CFD simulation of naked flame manikin tests of fire proof garments. Fire Saf. J..

[37] Lee C., Kim Y., Wood A. (2002). Investigation and correlation of manikin and benchscale fire testing of clothing systems. Fire Mater..

[38] Dukes-Dobos F.N., Reischl U., Buller K. (1992). Assessment of ventilation of firefighter protective clothing. ASTM Int..

[39] Rucker M., Anderson E., Kangas A., Nelson C.N., Henry N.W. (2000). Evaluation of standard and prototype protective garments for wildland firefighters. Performance of Protective Clothing: Issues and Priorities for the 21st Century.

[40] Ruckman J.E., Murray R., Choi H.S. (1998). Engineering of clothing systems for improved thermophysiological comfort: The effect of openings. Int. J. Cloth. Sci. Technol..

[41] Bouskill L.M. (1999). Clothing Ventilation and Human Thermal Response. Ph.D. Thesis.

[42] McQuerry M., Den Hartog E., Barker R. (2016). A review of garment ventilation strategies for structural firefighter protective clothing. Text. Res. J..

[43] Li X.H., Lu Y.H., Zhai L.N., Wang M., Li J., Wang Y.Y. (2015). Analyzing thermal shrinkage of fire-protective clothing exposed to flash fire. Fire Technol..

[44] Wang M., Li J. (2016). Thermal protection retention of fire protective clothing after repeated flash fire exposure. J. Ind. Text..

[45] Zhang X., Tian X., Yao X. (2008). Isothermal and non-isothermal shrinkage behaviors of highly oriented PET yarns. Fiber Polym..

[46] Pakula T., Trznadel M. (1985). Thermally stimulated shrinkage forces in oriented polymers. I. Temperature dependence. Polymer.

[47] Trznadela M., Kryszewskia M. (1992). Thermal shrinkage of oriented polymers. J. Macromol. Sci. Part C.

[48] Hindeleh A.M., Johnson D.J. (1978). Crystallinity and crystallite size measurement in polyamide and polyester fibres. Polymer.

[49] Torvi D.A., Dale J.D., Faulkner B. (1999). Influence of air gaps on bench-top test results of flame resistant fabrics. J. Fire Prot. Eng..

[50] Sawcyn C.M.J. (2003). Heat Transfer Model of Horizontal Air Gaps in Bench Top Testing of Thermal Protective Fabrics. Master’s Thesis.

[51] Li J., Li X.H., Lu Y.H., Wang Y.Y. (2012). A new approach to characterize the effect of fabric deformation on thermal protective performance. Meas. Sci. Technol..

[52] Dolez P.I., Tomer N.S., Yassine M. (2018). A quantitative method to compare the effect of thermal aging on the mechanical performance of fire protective fabrics. J. Appl. Polym. Sci..

[53] Torvi D.A. (1997). Heat Transfer in Thin Fibrous Materials under High Heat Flux Conditions. Ph.D. Thesis.

[54] Jain A., Vijayan K. (2002). Thermally induced structural changes in Nomex fibres. Bull. Mater. Sci..

[55] Arrieta C., David E., Dolez P., Vu-Khanh T. (2011). X-ray diffraction, Raman, and differential thermal analyses of the thermal aging of a Kevlar-PBI blend fabric. Polym. Compos..

[56] Deng M., Tian M., Wang Y.Y., Wang M. (2020). Quantitatively evaluating the effects of flash fire exposure on the mechanical performance of thermal protective clothing. Int. J. Cloth. Sci. Technol..

[57] Tian M., Li J. (2018). 3D heat transfer modeling and parametric study of a human body wearing thermal protective clothing exposed to flash fire. Fire Mater..

[58] McQuerry M., Klausing S., Cotterill D., Easter E. (2015). A Post-use Evaluation of Turnout Gear Using NFPA 1971 Standard on Protective Ensembles for Structural Fire Fighting and NFPA 1851 on Selection, Care and Maintenance. Fire Technol..

